# Development of a tool to measure women’s perception of respectful maternity care in public health facilities

**DOI:** 10.1186/s12884-016-0848-5

**Published:** 2016-03-29

**Authors:** Ephrem D. Sheferaw, Teka Z. Mengesha, Solomon B. Wase

**Affiliations:** Jhpiego an affiliate of Johns Hopkins University, 2881, code 1250 Addis Ababa, Ethiopia; Institute of Psychology, Addis Ababa University, Addis Ababa, Ethiopia

**Keywords:** Respectful Maternity Care (RMC), Disrespect and Abuse (D&A), Scale

## Abstract

**Background:**

Maternal mortality continues to be the biggest challenge facing Ethiopia and other developing countries. Although progress has been made in making maternity services available closer to the community, the rate of deliveries attended by skilled birth attendants has remained very low. Absence of respectful maternity care (RMC) is believed to have contributed to low utilization of facility delivery services. This study outlines steps undertaken to construct and validate a scale that measures women’s perception of respectful maternity care provided in health facilities.

**Methods:**

An inductive item generation process that included a literature review and in-depth interviews with labor and delivery clients, followed by an expert review, assured face validity and content validity of the tool. A draft RMC scale with 37 items and two additional measures of global satisfaction items, measured on a five-point Likert scale, were administered to a developmental sample of 509 postnatal care clients visiting facilities immediately after childbirth to 7 weeks postpartum. IBM SPSS 20 was used to perform exploratory factor analysis (EFA) using principal component analysis (PCA) with oblique rotation method.

**Results:**

The final RMC scale with 15 items was loaded on four components. The extracted components were labeled as friendly care, abuse-free care, timely care, and discrimination-free care. The final RMC scale correlated strongly with the global satisfaction measures, indicating criterion-related validity of the scale. Content-related validity was assured by the process of item generation. Construct validity of the RMC scale was confirmed by high average factor loading of the four components ranging from 0.76 to 0.82 and low correlation between the components. Stability of the scale was confirmed by running PCA in a randomly selected split sample of 320 samples from the validation sample. The final 15-item scale showed an adequate reliability with α = 0.845.

**Conclusion:**

The 15-item RMC scale is a valid and reliable measure of women’s perception of RMC received in health facilities. We recommend that health facilities use the RMC scale in urban public health facilities and that other researchers conduct further exploratory and confirmatory factor analysis in different geographic areas.

## Background

In 2013 about 289,000 women died, worldwide, due to complications in pregnancy and childbirth. Although maternal death has declined 45 % from the 1990 estimate, the number of deaths per year is still unacceptably high [1]. Maternal mortality, the death of a woman while pregnant or within 42 days of termination of pregnancy, continues to be the most formidable challenge for Ethiopia [2]. Ethiopia was one of the 10 high burden countries that accounted for 58 % of global maternal deaths from 1990 to 2013 [1]. The maternal mortality ratio for Ethiopia in 2011 was 676 per 100,000 live births, and the proportion of deliveries assisted by a skilled birth attendant was 10 % [2].

Reasons for low utilization of services at health institutions include 1) cultural barriers, 2) provider-client interpersonal barriers, 3) economic barriers, and 4) geographic barriers [3]. Similarly, a literature review conducted by the Maternal and Child Health Integrated Program (MCHIP) in Ethiopia showed that women’s perceptions about health facilities’ cleanliness, equipment quality or availability, provider competence, or behavior can be barriers to institutional delivery utilization. Some communities express dissatisfaction with providers’ medical advice or management [4].

A woman’s experience of care in childbirth is an important determinant of her future decisions related to seeking health care from health facilities. Women’s negative encounters with health workers during delivery can result in long-lasting damage and emotional trauma [5].

Generally, there is lack of agreement on a consistent definition of respectful maternity care. Even the term respectful maternity care has been used synonymously with women-friendly care and women-centered care. Respectful maternity care (RMC) encompasses the universal right of every childbearing woman to receive care that includes respect for the woman’s autonomy, dignity, feelings, choices, and preferences including choice of companionship and cultural rituals at birth in institutional delivery, whenever possible [6]. RMC is closely related to eliminating disrespect and abuse during pregnancy and childbirth [7, 8]. Based on a comprehensive desk review of the evidence, the following seven categories of disrespect and abuse in childbirth were identified: physical abuse, non-consented care, non-confidential care, non-dignified care, discrimination based on specific patient attributes abandonment of care, and detention in facilities [3].

The absence of respectful maternity care is recognized as a deterrent to utilization of maternity care services [3, 9]. Women’s level of satisfaction with maternity care is closely related to the way they are treated by health workers [10]. There are no reliable estimates of the prevalence of abuse and disrespect [3, 11, 12]. The majority of studies conducted in measuring respectful maternity care used qualitative approaches and structured interviews with dichotomous responses on single items.

Although there is an overall agreement that disrespect and abuse are important barriers to utilization of services at health facilities, there is still no generally agreed-upon operational definition of these terms [3, 8], and there is an urgent need for a validated assessment tool that can measure women’s perceptions of respectful maternity care that encourages women to use maternity care services. Constructing a tool will also help health facility managers to monitor their clients’ level of satisfaction with the services and make the necessary adjustments to address clients’ needs. The overall objective of this analysis was to construct a scale that measures women’s perception of respectful maternity care provided in public health facilities of Ethiopia and determine its reliability and validity.

## Methods

### Study population and sample

The study was conducted in 11 urban-based public health facilities (three hospitals and three health centers in Addis Ababa, one hospital and one health center in Bishoftu, and one hospital and two heath centers in Adama town). This population, which was used for developing and validating the scale, is referred to as the developmental group. The target population for this study consisted of postpartum women who delivered in public health facilities within seven weeks prior to data collection.

### Study design

The study utilized a mixed approach of qualitative and quantitative methods. The qualitative approach used in-depth interviews with postpartum women. In the quantitative approach, expert review was undertaken by trained data collectors using email and interviews with postpartum women.

The study was conducted in three phases. First, a formative phase was carried out to determine potential items that could be included in the tool. This initial phase included a comprehensive literature review followed by in-depth interviews with eight postpartum women in two health facilities. In the second phase, the draft items were pilot tested among 40 postpartum women in five health facilities. In the third phase, a quantitative assessment was conducted in a private area within the selected health facilities. Postpartum women interviewed were those who received labor and delivery services in public health facilities within 7 weeks prior to the date of the interview, consented to participate in the study, and visited health facilities during the data collection period.

### Sampling

A consecutive sampling approach was utilized. In-depth interviews with eight postpartum women helped to saturate RMC dimensions. For piloting the draft tool, interviews with 40 postpartum women were conducted. The probability that a factor structure can be replicated in another study depends partially on the sample size used in the initial analysis [13]. For the final administration of the RMC tool, sampling recommendations, stated in terms of the ratio of a minimum sample size (N) for a particular analysis to the number of variables (p), were used [14, 15]. Tinsley and Tinsley (1987), cited by DeVellis (2003), suggest that proportions of 5 to 10 subjects to one variable is sufficient [16]. An empirical test conducted by Costello and Osborne on the effect of sample size on the results of factor analysis reported that larger samples tend to produce more accurate solutions [13].

In this study, the number of variables (p) was 37 items, and a total of 509 women (N) were interviewed, which resulted in nearly 14 subjects to one variable.

### Data collection

Data were collected from postpartum women during March 2014 in the 11 health facilities across three cities. The interviews were conducted at intervals ranging from 6 h to 7 weeks after delivery. Forty-two percent of mothers were interviewed within 2 days of delivery, 14 % were interviewed from 3 to 42 days after delivery, and the remaining 44 % were interviewed from 43 to 49 days after delivery. Inclusion criteria for women were as follows: use of delivery services in public health facilities from 6 h to 49 days before data collection, ability to speak Amharic, and willingness to participate in the study.

To avoid professional bias during data collection, we selected experienced non-health professional (information technology and social science background) data collectors. The principal investigator and co-investigator supervised the data collection process.

### Procedures

**Fig. 1:**
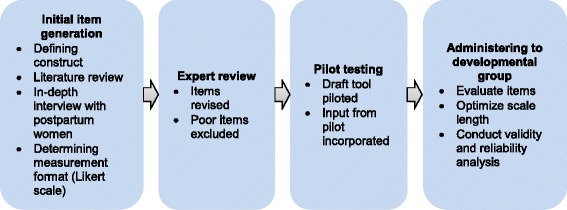
RMC tool development process

RMC tool development was conducted using psychometric procedures recommended by DeVellis (2003) on procedures for new scale development [16]. This included initial item generation, expert review, pilot testing, and final administration of the draft tool to the developmental group. Each step of the development process is described in Fig. 1 and in the next section.

### Initial item generation

The literature review identified seven a priori dimensions. In each of these, 5–12 items were selected from the pool of items generated by in-depth interviews conducted to understand the perception of care received by eight postpartum women during the delivery and postnatal periods. This resulted in a draft tool, or scale, with 60 items. A five-point Likert scale (with 5–strongly agree, 4–agree, 3–I don’t know, 2–do not agree, and 1–strongly do not agree) was used.

### Expert review

The 60-item draft scale was reviewed by five maternal and newborn health experts in Ethiopia. The experts were all public health practitioners with masters’ degrees in public health as well as a bachelor’s degree in midwifery, public health, or medicine. These experts had from 10 to 35 years of experience in teaching, program management, and clinical work related to maternity care. All experts who participated were executive board members in their respective associations. These associations included the Ethiopian Public Health Association (EPHA), Ethiopian Public Health Officers association, Ethiopian Midwifery association, and Ethiopian Evaluation association. Using the comments of experts, four items were excluded, five items rephrased, and three new items added.

### Pilot testing

After incorporating experts’ feedback, the 59-item draft scale was random ordered and formatted to use in a pilot test conducted among a sample of 40 postpartum women in two hospitals and three health centers in Adama and Addis Ababa. The findings guided several changes to the tool: three items were merged into one and 20 items (those that were not clear to respondents or were redundant) were excluded, resulting in a tool with 37 items for final administration to the developmental group.

### Data collection from the developmental group

Data were collected in March 2014. All data collectors received a half-day orientation on administration of the scale, the informed consent process, confidentiality of data, and the role of the data collector during the survey. Data collectors presented a support letter, obtained from the regional health bureaus of Addis Ababa and Oromia for the 11 facilities, to health facility managers and maternity unit coordinators to inform them of the study objectives. After this, the data collectors began their work. All women who used labor and delivery services in public health facilities within 49 days preceding the survey were invited to participate. Informed consent was requested and obtained for all women.

To maintain the women’s privacy, all interviews were conducted in a private area inside the health facility. Interviews with immediate postnatal clients were conducted in the postnatal room when health providers were not around and no other mothers were in the room.

### Data entry

After the data collected were reviewed for completeness, data entry was conducted. Data entry was managed using a data entry template prepared in Microsoft Access 2010. Double data entry was used for 25 % of cases and validated with the original and no discrepancy was obtained to proceed to 100 % double entry.

### Data analysis

**Fig. 2:**
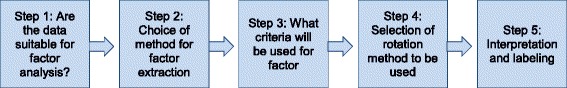
Five-step data analysis process

Data analysis was performed using the IBM SPSS 20 statistical package. Data analysis followed steps for new scale construction outlined by Worthington and Whittaker (2006) [15] and DeVellis (2003) [16]. The steps are outlined in Fig. 2.

Exploratory factor analysis (EFA) using a principal component analysis (PCA) was used to identify a parsimonious list of factors that describe women’s perception of RMC and consolidate variables and generate hypotheses about underlying processes [13]. The Kaiser-Meyer-Olkin (KMO) measure of sampling adequacy and Bartlett’s test of sphericity were used to check the suitability of data for factor analysis. The reliability of each component was assessed using Cronbach’s alpha.

Correlation analysis and independent samples t-tests were used to assess validity of the tool with criterion satisfaction items and other background information of women and type of delivery.

### Factor analysis

Establishing dimensionality of a construct is an important step in the scale development process [17]. In this analysis a KMO value of 0.6 was used as the criterion for sampling adequacy. To produce scale uni-dimensionality and simplify the factor solutions, scree plot and parallel tests were used as criteria for factor extraction.

Rotation is a statistical technique used to simplify interoperability of factor solution [13]. Oblique rotation was used as a method of rotation. Use of oblique rotation was justified because RMC components are closely correlated. The rotation was conducted in a series of seven iterative processes, deleting one or more items at a time and examining the remaining items.

Item loading (which refers to the degrees to which the original item scores correlate with the components), cross loading, and communalities were used as criteria for item deletion. If factors shared items that cross-loaded too highly on more than one factor (e.g., > 0.32) or if factors shared items that cross-loaded and the difference in item loading from the highest was less than 0.15, it was rejected. Communalities (the amount of variance of a measure that is accounted for by a component or group of components derived from factor analysis before rotation) was the third criterion, where item communalities of less than 0.6 after rotation were used as the lowest limit for item deletion. Cross loading was not used as a pragmatic statistical criterion for item deletion; instead, the judgment of the researcher and study team members was used to delete or retain items to relevant factors based on their theoretical significance.

### Validity and reliability analysis

Evidence about different forms of validity of RMC components was obtained using PCA. This section describes how the evidence for different forms of validity was inferred.

*Content-related validation* involved assessing the degree to which the sample of items, tasks, or questions on a test is representative of some defined universe or domain of content, based on expert judgment. Face validity, which is closely related to content validity and refers to whether a measure appears to be measuring what it is supposed to measure, was also assessed [10, 18].

*Criterion-related validation* consisted of verifying whether a test score on the scale was correlated with criteria measured at the same time. This is usually based on comparison between an existing scale and the one under development, but in our case, no appropriate scales existed for the construct. Therefore, we selected two criteria using the experiences of other researchers on a closely related variable: satisfaction with overall service and recommendation to others [10].

*Construct validation* relates to how well the items on a questionnaire represent the underlying conceptual structure. Construct validation was assessed by examining the Pearson correlation coefficient between components identified by factor analysis. Known-groups validity (also a form of construct validity) was ensured by assessing the scale’s ability to differentiate the level of RMC reported for normal and complicated deliveries.

*Reliability analysis* was used to assess the internal consistency of the scale. The internal consistency of each component of the RMC scale was assessed using Cronbach’s alpha. To be considered consistent, the minimal coefficient for a component had to be above 0.70 [10].

### Ethical consideration

The proposal for this study was reviewed and approved by the Addis Ababa University Faculty of Education and Behavioral Sciences ad hoc research ethics committee, the Addis Ababa Regional Health Bureau institutional review board, and the Oromia Regional Health Bureau institutional review boards. All women interviewed were asked for their informed consent to participate.

## Results

### Characteristics of mothers surveyed

A total of 515 recently delivered women responded to the survey across the 11 health facilities. Six of the questionnaires were excluded due to incompletion. All of the remaining 509 respondents were included in the analyses. Residence of these respondents was predominantly urban (95.1 %). The average age was 27.4 years with a standard deviation of 4.8, minimum 16 years and maximum 46 years. The women’s parity ranged from zero to six; 51 % had a spontaneous vaginal delivery; 12.3 % had a cesarean section; and 36.7 % had an episiotomy.

In-depth interviews conducted with postpartum women showed that some forms of abuse and disrespect were prevalent in the study area. The in-depth interview participants reported some form of physical abuse. A 20-year-old mother reported that, while she was having pain during labor, she touched a young female health worker and the health worker threw the mother’s hand away. Incidents such as this shocked the women and caused them to lose trust in the health workers. Women also reported that non-consented care has occurred so frequently that mothers do not expect to be asked for their consent for all procedures. A 23-year-old mother reported that, after she was referred from a health center and had waited for some time at a teaching hospital, students came one after the other, asked her to open her legs, and inserted their fingers into her vagina without explaining the reason. In-depth interview participants also reported non-dignified care. Examples of this type of care included health workers shouting at women for not attending antenatal care services and for making noises during their labor pains. A 20-year-old woman reported discrimination during hospital referrals. She was referred to hospitals where she was not able to get services and was later referred to another hospital. Delays caused by these referrals were painful for her, and she felt the health workers should have prioritized her. The 20-year-old woman reported abandonment during her stay at a health center, but detentions in facilities were not reported because the government provided delivery services free of charge.

### Factor analysis

Suitability of data for factor analysis was confirmed by KMO and Bartlett’s test of sphericity. The KMO value for RMC scale was *0.903*, indicating that there are components in the correlation matrix to uncover. Bartlett’s test of sphericity, which was *χ*^2^ (666) = 9229, *p* < 0.0001, indicated that correlation between the items was sufficiently large for PCA. The two tests indicated that use of PCA was appropriate.

The scree plot suggested four components explaining *50.3 %* of the variation in the initial 37-item scale solution and 67.8 % of the final 15-item scale. Parallel analysis, using Monte-Carlo PA software-generated random data, also confirmed four components. Multidimensionality of the RMC construct was confirmed by the diagnostic tests.

Oblique rotation (direct oblimin with Kaiser normalization) in a series of iterative processes produced four components with eight, three, three, and two items on the four components, respectively.

Table 1 below show items deleted for different reasons.

**Table 1:** Items deleted during analysis for different reasons, 2014

Items	Reason for deletion
Q202 Some health providers showed me an intimidating gesture (R)	Low communalities
Q206 I was left alone after delivery for a long time (R)	
Q203 The counseling sessions were held in a private area	
Q234 The health workers provided coaching on breathing and relaxation	
Q204 The health workers talked to me and my companions politely	
Q201 The health provider greeted me and my companions before service delivery	
Q209 The health worker didn’t mention anything that he/she was performing (R)	
Q231 The health workers showed active involvement during contraction	
Q236 I felt like the health workers tried to move things along for their own convenience (R)	
Q221 The health worker encouraged me to open my legs during labor	
Q210 I was detained in the facility because I didn’t have enough money to pay for the service I was given (R)	Low factor loading
Q213 The couches were separated by privacy screens during examination	
Q215 Some health workers do not treat all patients equally (R)	High cross loading on two or more factors
Q217 My consent was requested for all procedures performed	
Q226 The health provider helped me to try different delivery positions	
Q229 During delivery, the health worker draped or covered me to protect my privacy	
Q230 The health workers used a reassuring touch	
Q220 My companions were allowed to enter the delivery room during delivery	
Q212 I was told that I can refuse a procedure if I don’t like it	
Q223 All health workers treat patients equally	
Q214 The health workers shouted at me for different reasons during contraction (R)	Decrease cross-correlation
Q235 I felt there was inappropriate touching of genitals/thighs during the exam (R)	

(R): Items are reverse coded

During the extraction process, 10 items were deleted for low communalities, eight items were eliminated for high cross loading on two or more factors, two items were eliminated due to low factor loading, and two items were deleted because they contributed to a decrease in cross correlation of other items.

Table 2 below shows the pattern matrix, which is the correlation between each item and uncorrelated components extracted after an iterative process of oblique rotation.

**Table 2:** Pattern matrix RMC scale, 2014

RMC Items	Components				Communality	Component label
	1	2	3	4		
Q232 I felt that health workers cared for me with a kind approach	0.811				0.724	Friendly care
Q211 The health workers treated me in a friendly manner	0.792				0.669	
Q233 The health workers talked positively about pain and relief	0.789				0.604	
Q237 The health worker showed his/her concern and empathy	0.777				0.677	
Q227 All health workers treated me with respect as an individual	0.731				0.632	
Q205 The health workers spoke to me in a language that I could understand	0.724				0.598	
Q207 The health provider called me by my name	0.703				0.599	
Q224 The health worker responded to my needs whether or not I asked		0.826			0.725	Abuse-free care
Q208 The health provider slapped me during delivery for different reasons (R)		0.820			0.765	
Q238 The health workers shouted at me because I haven’t done what I was told to do (R)		0.781			0.725	
Q216 I was kept waiting for a long time before receiving service (R)			0.897		0.743	Timely care
Q225 I was allowed to practice cultural rituals in the facility			0.710		0.587	
Q219 Service provision was delayed due to the health facilities’ internal problem (R)			0.684		0.666	
Q222 Some of the health workers did not treat me well because of some personal attribute (R)				0.840	0.76	Discrimination-free care
Q218 Some health workers insulted me and my companions due to my personal attributes (R)				0.820	0.718	

(R): Items are reverse coded

Extraction method: Principal component analysis

Rotation method: Oblimin with Kaiser normalization

The four components of the 15-item scale extracted by PCA were labeled as friendly care, abuse-free care, discrimination-free care, and timely care considering the core idea explained by the predominant items in terms of factor loading in each subscale [19].

The 15-item RMC scale’s mean score for the developmental group was 57.83 with standard deviation of 8.46. The mean and standard deviation for each component were 28.54, 5.18 for friendly care; 10.87, 2.98 for abuse-free care; 9.90, 2.78 for timely care; and 8.52, 1.59 for discrimination-free care.

Analysis of inter item consistency showed good internal correlation with Cronbach’s alpha of 0.857 for standardized items for the full 15-item scale: 0.889 for friendly care, 0.75 for abuse-free care, 0.71 for timely care, and 0.666 for discrimination-free care.

Content validity of the RMC scale was assured through a methodological rigor that included review of related literature, in-depth interviews with postpartum women, and expert review.

Concurrent validity was assessed by correlating RMC scale with global satisfaction criterion items (Q339–satisfied with overall service and Q340–recommend facility to others). Pearson product moment correlation of summated RMC scale score with Q339 and Q340 showed a correlation coefficient of 0.711, *p* < 0.001, and 0.881, *p* < 0.001, respectively.

Construct-related validity was confirmed by examining the components correlation matrix for the rotated final components. This indicated a minimal correlation among components as shown in Table 3 below. Small Pearson correlation coefficients between components were observed.

**Table 3:** Component correlation matrix RMC scale, 2014

Component	Friendly care	Abuse-free care	Timely care	Discrimination-free care
Friendly care	1.0	0.113	0.356	0.250
Abuse-free care	0.113	1.0	0.019	0.065
Timely care	0.356	0.019	1.0	−0.009
Discrimination-free care	0.250	0.065	−0.009	1.0

Principal component analysis on a random split sample (320 out of the 509 samples) of the developmental sample confirmed factor stability; all four factors were retained with minimal change in factor loading. The scale accounted for 68.8 % of the variation as compared to 67.8 % in the full scale.

## Discussion

Review of the draft tool by a panel of maternal and newborn health experts improved the content coverage as well as the relevance of items in identified dimensions to local contexts and addressed face validity and content validity of the scale. Evidence for construct validity of the scale was obtained from factor analysis, which showed stability of the four components (friendly care, abuse-free care, timely care, and discrimination-free care) and also good internal consistency. The observed reliability falls in the range of acceptable internal consistency described by DeVellis (2003) [16].

In addition, correlation between components showed low correlation coefficients among the four components, which is considered to be strong evidence for construct-related validity. The evidence related to construct validity of the RMC construct implies that the identified components (friendly care, abuse-free care, timely care, and discrimination-free care) are the four dimensions that represent the perception of RMC provision in the public facilities examined in Ethiopia.

Concurrent validity was ensured by correlating the summated RMC tool with two items included in the tool that measured global satisfaction; these two items were considered to be closely related to RMC (satisfaction with overall service and recommend facility to others). A strong correlation coefficient of the two items (0.711, *p* < 0.001 and 0.881, *p* < 0.001) indicated evidence for concurrent validity. The strong correlation between the RMC scale and global satisfaction measures indicates that women who are satisfied with labor and delivery services also show a higher rating for RMC services.

### Strength

This study is one of the first studies on tool development for RMC that uses quantitative methods with multiple items. The majority of studies on RMC were conducted using qualitative approaches or single-item quantitative methods, which pose questions about validity and reliability. The seven dimensions of RMC identified by Bowser and Hill (2010) [3] were based on desk review.

This work can pave the way for other researchers to explore the construct further and produce other tools. The study utilized psychometric recommendation, which helped to produce a tool that has content and construct validity and good internal consistency.

### Limitation

For this study the developmental groups were selected from public hospitals and health centers in three towns in Ethiopia. To use this tool, additional studies need to be conducted by including rural hospitals and health centers. Some of the important dimensions of RMC identified by literature review (consented care, confidential care, and non-abandonment) were not identified. Although experiences of consented care and confidential care were identified in the in-depth interviews, the EFA process could not extract this component. This calls for further exploratory work in RMC using a different sample.

## Conclusion

The 15-item RMC scale developed with four components (friendly care, abuse-free care, timely care, and discrimination-free care) was found to be a valid and reliable measure of women’s perception of respectful maternity care provided in public health facilities based on the information found from the developmental group. The factor structure of RMC needs to be further confirmed by additional exploratory and confirmatory factor analysis studies in additional sample areas.
